# Integrated multi-level quality control for proteomic profiling studies using mass spectrometry

**DOI:** 10.1186/1471-2105-9-519

**Published:** 2008-12-04

**Authors:** David A Cairns, David N Perkins, Anthea J Stanley, Douglas Thompson, Jennifer H Barrett, Peter J Selby, Rosamonde E Banks

**Affiliations:** 1 Clinical and Biomedical Proteomics Group, Cancer Research UK Clinical Centre, Leeds Institute of Molecular Medicine, St James's University Hospital, Beckett Street, Leeds LS9 7TF, UK; 2 Section of Epidemiology and Biostatistics, Leeds Institute of Molecular Medicine, St James's University Hospital, Beckett Street, Leeds LS9 7TF, UK; 3 Department of Clinical Biochemistry and Immunology, Leeds General Infirmary, Great George Street, Leeds LS1 3EX, UK

## Abstract

**Background:**

Proteomic profiling using mass spectrometry (MS) is one of the most promising methods for the analysis of complex biological samples such as urine, serum and tissue for biomarker discovery. Such experiments are often conducted using MALDI-TOF (matrix-assisted laser desorption/ionisation time-of-flight) and SELDI-TOF (surface-enhanced laser desorption/ionisation time-of-flight) MS. Using such profiling methods it is possible to identify changes in protein expression that differentiate disease states and individual proteins or patterns that may be useful as potential biomarkers. However, the incorporation of quality control (QC) processes that allow the identification of low quality spectra reliably and hence allow the removal of such data before further analysis is often overlooked. In this paper we describe rigorous methods for the assessment of quality of spectral data. These procedures are presented in a user-friendly, web-based program. The data obtained post-QC is then examined using variance components analysis to quantify the amount of variance due to some of the factors in the experimental design.

**Results:**

Using data from a SELDI profiling study of serum from patients with different levels of renal function, we show how the algorithms described in this paper may be used to detect systematic variability within and between sample replicates, pooled samples and SELDI chips and spots. Manual inspection of those spectral data that were identified as being of poor quality confirmed the efficacy of the algorithms. Variance components analysis demonstrated the relatively small amount of technical variance attributable to day of profile generation and experimental array.

**Conclusion:**

Using the techniques described in this paper it is possible to reliably detect poor quality data within proteomic profiling experiments undertaken by MS. The removal of these spectra at the initial stages of the analysis substantially improves the confidence of putative biomarker identification and allows inter-experimental comparisons to be carried out with greater confidence.

## Background

Clinical proteomic profiling experiments using high throughput mass spectrometry (MS) technologies such as matrix assisted laser/desorption ionisation time-of-flight mass spectrometry (MALDI-TOF-MS) and the derivative surface enhanced laser desorption/ionisation time-of-flight mass spectrometry (SELDI-TOF-MS) have provided encouraging and exciting results over the past few years [1-4]. However, many studies have been the subject of debate, both in terms of the reproducibility of results and regarding issues of technical and experimental design [5-7]. A common criticism has been the difficulty in identifying the protein or peptide signified by the specific informative peak(s) in the mass spectrum, and indeed if it has been possible, then the identified protein has often been of relatively high abundance and not disease-specific [8].

There is now an expanding literature which describes good practice in undertaking such experiments. Reanalysis of published data has raised a number of important issues relating to experimental design and dry-lab (statistical and bioinformatic) analysis of experimental results [9-12]. Sample size calculations have been developed to allow the undertaking of statistically powerful experiments [13] and a number of sample handling issues in sample collection [14] and pre-fractionation [15] have been investigated. Spectral pre-processing methods such as calibration [16,17], spectral alignment [17-19], baseline subtraction [16,20,21], normalisation [22,23] and peak detection [24-26] have been critically evaluated and developed broadly in line with published recommendations for proteomic analysis [7,27]. Other studies have highlighted the problems of limited reproducibility and transferability of discoveries to larger multi-site validation studies [28,29]. However, a somewhat neglected step is the quality control (QC) of proteomic profiling experiments using MS. The importance of experimental monitoring and QC has been appreciated in other procedures, e.g. in sequence interpretation [30] as well as to some extent in the setting of mass spectrometric proteomic profiling experiments [10,25,31,32]. Additionally, some evaluation of the sources of variation in the experimental procedure has been undertaken [33]. However, the QC tools available in the literature, e.g. [34,35] do not fulfil all of the requirements of a QC strategy that is integrated to the laboratory and will perform both continuous monitoring of the experiment over time [36] and also allow similarity analysis of technical replicates. In common with many analytical procedures, technical replicates in addition to biological replicates are standardly included in such studies due to the inherent variability in the experimental technique. QC of such replicates allows reproducibility to be assessed while their inclusion implicitly reduces the impact of technical variation.

The objective here is to present a web-based tool for QC of proteomic profiling experiments undertaken using MS. Such a tool should be easily integrated into the data management tools which are included with an instrument. Factors that can affect an MS profile include, time (since first determination), temperature, humidity, the instrument used and the laboratory [10], residual potentials on the deflection plates after the deflection pulse, plate planetary imperfections, pipetting errors, matrix crystallisation [17], laser/detector deterioration, and in the case of SELDI – variability in chip surface [25]. Experiments should be undertaken to assess the magnitude of each of these factors so the system is well understood. Such a list clearly demonstrates the need for a QC tool to be multi-facetted, i.e. it should be able to monitor the performance of the experiment throughout an experimental run for unusual variation which could be caused by machine, chromatographic separation or operator malfunction to provide an early alert. Additionally, analysis of replicates to identify poor reproducibility and hence the need for further technical replicate determinations of a particular sample should be integral. Our system fulfils each of these requirements and we demonstrate its use using data from a proteomic profiling study of patients with different levels of renal function undertaken using SELDI-TOF-MS. Although the system described here is using SELDI-TOF, the method is designed so that it can be easily extended to MALDI-TOF instruments generally and indeed to similar data from other current and future technological platforms, i.e. two-dimensional spectral data. As a secondary theme we investigate the variation attributable to different factors in the experiment (biological variance and technical variance due to day/chip/spot/other) using data from a profiling study of renal function, together with specific examples from other studies.

## Methods

### Biological samples and profile generation

The main study set used was data generated from a comparison of serum samples collected from patients with renal failure prior to dialysis (*n *= 30), two groups of patients post-renal transplantation with stable (*n *= 30) and unstable (*n *= 20) renal function and normal healthy controls (*n *= 30) with similar age and gender distributions to the clinical groups [37]. All samples were processed within 1 hour of venepuncture according to a standardised operating procedure and stored at -80°C. Details of the preparation of CM10 (weak cation exchange) and IMAC-Cu (immobilized metal ion affinity chromatography) sample chips and the application of saturated sinapinic acid solution (Fluka) using the Biomek robot are as described previously [14]. Low mass acquisition was between 2–10 kDa, focussed at 6 kDa, with laser intensity set at 1800 nJ and medium mass acquisition was 10–20 kDa, focussed at 15 kDa, with laser intensity of 2200 nJ. Samples were spotted in duplicate on ProteinChips following complete randomisation throughout all replicates. A QC sample was formed by pooling serum from all individuals in the study. The QC sample was aliquotted to avoid freeze-thaw cycles and all aliquots stored at -80°C until used in the QC protocol is described in Section 2.2. The initial part of the study took three days for each chip type with chips 1–13 being processed on day 1, chips 14–24 on day 2 and chips 25–33 on day 3. Any subsequent repeated technical replicates were undertaken one week later in singlicate.

For calibration purposes, an H50 chip spot was loaded with 2 μl of matrix containing 400 fmol of each of bovine insulin (Mr 5733.58), bovine ubiquitin (Mr 8564.8), bovine Cytochrome C (Mr 12230.92), equine cardiac myoglobin (Mr 16951.51) and bovine β lactaglobulin A (Mr 18363.3) calibrants. After air-drying, the calibrant mixture was analyzed on the SELDI PCS4000 Enterprise system (Ciphergen, Fremont, CA) using standard acquisition parameters for the low and medium mass ranges. Calibration was performed using single and double charged peaks where appropriate and a quadratic calibration equation generated for use in the study using Ciphergen Express 3.0 (Ciphergen, Fremont, CA). A stock calibration solution was prepared at the start of the study to calibrate the machine. Subsequently, a fresh calibrant spot was prepared and analysed each day to check for any calibration drift by viewing plots of calibration spectra [24]. If any gross changes were observed a new calibration equation was generated and applied to subsequent samples, but generally recalibration was not required day-to-day.

### QC protocol

The QC sample was central to both facets of QC – the monitoring of performance throughout the study and also the analysis of technical replicates. The QC sample was formed by pooling serum from all individuals in the study. This is good practice as creating a QC sample from just a few samples can allow peaks in a few dominant samples to make the QC sample unrepresentative of the majority of samples in an experiment (data not shown). The QC sample was spotted onto three ProteinChip arrays (24 spots) at the beginning of the run on the first day of the analysis to define a "reference set" which was used to characterise normal within-run technical variability in the profiling technique and then allowed the assessment of future experiments using this reproducible profile. To assess between-chip and between-day technical variation, the QC sample was also included on a single spot on each ProteinChip used in the analysis, ensuring equal use of spots A to H for this purpose. This protocol is shown diagrammatically in Figure S1 [see Additional file 1] for this experiment which consists of 35 chips in total. All of the QC samples were used in defining limits of variability in replicate analysis. In following sections the 3 ProteinChips which contained only the QC samples and define the reference set are referred to as QC chips for brevity. Similarly, sample chips refer to the other ProteinChips in the study which contain only one spot of the QC sample and the remaining spots samples from the full study.

### Data storage, extraction and pre-processing

The spectral data produced by the SELDI-TOF-MS are standardly stored in a MySQL relational database supplied by Ciphergen. Raw intensity values produced by the detector are stored as a simple binary blob consisting of integer values which are multiplied by a constant (also stored within the database) to produce actual intensity values whereas mass values are calculated using the quadratic calibration equation. All this data is retrieved from the SQL database by query functions utilising the MySQL C application programming interface (API) with no further use being made of the Ciphergen software.

Each SELDI experiment has associated with it a list that identifies the appropriate spectra along with information defining the nature of each sample (i.e. sample replicate or pooled QC sample). This list is uploaded via a web browser and is used to identify the data that should be extracted from the SQL database as described above. This data then undergoes pre-processing steps (baseline subtraction, internal normalisation for QC and peak detection [see Additional file 2]) before being passed to the statistical routines described in the next section. However, it should be noted that we do not use this (or any other form of) normalisation in analysis of the baseline subtracted and peak detected profile in any post-QC analysis.

### Statistical methods

The QC procedure described here provides two functions. The first of these is to monitor the profiles being generated in a study over time by comparing the QC profile from sample chips with those defined by the QC chips, i.e. the reference set. The second is to evaluate the similarity of replicates and make decisions on whether further technical replicates are required. This was undertaken by comparing various parameters from the spectra on the sample chips with the corresponding parameters in QC spectra. In the following two sub-sections brief details are given of the methodology that underlies these procedures.

#### Chip-to-chip QC

Chip-to-chip QC was undertaken by using a transformation of data from the reference set and then comparing new QC spectra from sample chips (transformed suitably) with this reference set. The transformation chosen is principal components analysis (PCA) [38], a vector space transform which is often used to reduce multidimensional data sets to lower dimensions for exploratory data analysis. Hence, the peak detected profile of the samples in the reference set were transformed into a set of composite variables which have the property that the first principal component (PC) has largest variance, the second PC has second largest variance and so on to the last PC which has the smallest variance. Plots of the first few PCs (generally all pair-wise combinations of PC1, PC2 and PC3) and dendrograms displaying hierarchical clustering using Ward's agglomeration method and the Euclidean distance metric were then examined for any observable clustering according to ProteinChip, spot or order of profile generation and also for obvious poor quality spectra (outliers in PC plots) which should be excluded from the reference set.

QC spectra from subsequent sample chips were then projected into the space defined by the PCs calculated on the reference set using the loadings from the PCA and the intensities from the peak detected profiles of these new QC spectra. Note that the QC spectra from the reference set and the QC spectra from the sample chips are peak detected together each time a new spectra is brought to QC. This allows new peaks that appear in subsequent spectra, but were not present in the reference set to be included in the procedure. These projected versions of the QC spectra from sample chips are then viewed in plots alongside the reference set to check for systematic bias due to chip, spot or order of sample generation.

In addition to the visual examination of the QC spectra from sample chips, a significance test based on the Mahalanobis distance (MD) of the transformed QC spectra from the centre of the PC space defined by the reference set is also constructed. This test calculates the MD,

MD = √ (**x-μ**)^**T **^**Σ**^-1^(**x-μ**),

where **x **is the projection of the QC spectra, **μ **is the mean vector (in this case the zero vector, **0**) and **Σ **is the covariance matrix for the PC space. A significance test is then performed under the null hypothesis that the MD from the origin of the PC space to the new QC spectrum is zero against the alternative hypothesis that it is not. This significance test is possible as it is known that the null distribution is a χ^2 ^distribution with *p *degrees of freedom under multivariate normality [39]. The value of *p *in this case is equal to the dimensionality of the PC space used in the test. This is determined as the number of PCs that are needed to explain at least 90% of the variance which is present in the full reference set. As these QC samples were all the same, we would expect that the first few PCs would explain the vast majority of the variation.

The first few PCs are sensitive to outliers that inflate variances or covariances (or correlations, if the PCA has been conducted in terms of the sample correlation matrix, rather than the sample covariance matrix) [40]. So by viewing plots of the PCs and testing whether the MD from the origin of the PC space is significantly different from zero, a QC spectrum can be assessed in the sense of whether it is similar to those obtained in the QC reference set.

The calculations in this analysis were undertaken using matrix algebra, prcomp() and plotting functions in the R software environment for statistical computing (R Development Core Team, Vienna, Austria).

#### Replicate analysis

A critical part of QC is identifying differences between technical replicates of individual samples in a study which are not similar enough to be carried forward to subsequent analysis. The analysis of technical replicates was undertaken by comparing various parameters which summarize the duplicates with the parameter values obtained from all possible pairs of duplicates in the reference set. The parameters considered were:

• the total ion current – the sum of all the ion signals in a mass spectrum over time [41] and hence equivalent to the area under the spectrum,

• the normalised total ion current – equivalent to above, but internally normalised to the values (0,1) within each spectrum,

• the total intensity of peaks – the sum of intensity of all peaks identified in a smoothed spectrum (stage 2 of the peak detection process described above) and

• the total number of detected peaks – the total number of peaks in a spectrum (not to be confused with the number of common peaks that make up peak clusters).

Additionally, as a further summarizing variable the difference between the intensities of each common peak in the pair of technical replicates was also calculated. This analysis was undertaken in segments of the total mass range being considered as this was found to make the QC more sensitive. Experience from a number of studies of different diseases using various sample types has shown that four equally sized mass segments provided an adequate balance of QC sensitivity as compared with computing time (data not shown).

In order to decide which technical replicates required further examination, the coefficient of variation (CV) was calculated between each of the parameters for the duplicate samples and then this was compared to the distribution of CVs for all possible pairs of spectra from the reference set containing all QC spectra (which had passed chip-to-chip QC). A parameter was flagged if the CV of the sample pair was greater than the 95% quantile of the distribution of CVs from the reference spectra, indicating that the pair of spectra should be examined, but not necessarily rejected. A greater number of flags indicated more differences between technical replicates and a greater likelihood of rejection of spectra upon examination.

The difference between intensities of common peaks in the technical replicates was compared with the reference set in a similar manner. Firstly, all the data (QC samples and study samples) were peak-detected together to define common peak clusters for all spectra. All possible pairs of the QC spectra were then considered and the absolute difference in intensity calculated at each peak cluster. The 95% quantile of these differences was then calculated for each peak cluster and this was used as a critical value to indicate whether the difference between replicates at that peak cluster is larger than it ought to be with reference to the QC samples. The percentage of peaks where the difference was larger than this critical value in each pair of technical replicates was then reported.

#### Variance components analysis

The lmer() function in the lme4 package [42] in R and OpenBUGS [43] were used to estimate nested variance components from the profiles in the full study which had passed QC, i.e. this is based on the study samples (subjects on dialysis, subjects post renal transplantation and healthy controls in duplicate) and not the QC samples, and includes replicates that were re-run post-QC. The variance components are nested because of hierarchical structure in the data arising from the fact that each chip (and hence each spot) is run on only one day. This is analogous to the nesting of technical replicates within biological replicates in other studies [44]. Of course the technical replicates of the biological replicates are not necessarily nested within the day and chip in this case. However, another level of nesting which is present is the nesting of duplicate technical replicates within the class of subjects.

Both formulations of the variance components model assumed normally distributed data and hence a normal likelihood. The Bayesian formulation of the variance components specified vague prior distributions for all terms using normal distributions with large variance. The classical estimation procedure used maximum likelihood estimation whereas the Bayesian method used Markov chain Monte Carlo [45], specifically Gibbs sampling [46], to sample from the posterior distribution. For each peak 3 chains of 25000 samples were drawn from the posterior distribution with the first 5000 samples discarded as burn-in. Convergence of the sampling scheme was evaluated using the scale reduction factor [47]. The marginal medians from the posterior distribution were then used as parameter estimates in the construction of tables and figures.

### Data presentation

The results of the QC analysis are presented to the user in the form of a web page with both graphical and tabulated data as described in the following sections. Links are also provided to a web based spectra viewing tool which allows multiple spectra to be plotted together facilitating manual scrutiny of any significant differences identified.

## Results and discussion

The experimental run was managed over three consecutive days and resulted in 110 samples of serum from subjects being realised as 220 proteomic profile spectra (i.e. duplicate technical replicates) and 56 spectra of the pooled QC serum sample (24 in the reference set from the first three chips and 32 on subsequent sample chips). The 24 results in the reference set were visually examined and decided to be grossly consistent prior to the commencement of the profiling of the rest of the samples. In the low mass range 267 common peaks were detected over the full mass range with (109, 70, 54, 34) found in the equally spaced quarters or mass segments of the region (2–4 kDa, 4–6 kDa, 6–8 kDa, 8–10 kDa). Similarly in the medium mass range there were 151 peaks with (45, 36, 34, 36) in the mass segments spanning (10–12.5 kDa, 12.5–15 kDa, 15–17.5 kDa, 17.5–20 kDa). In the following sections the results of QC undertaken at the end of the third day is demonstrated, but it should be noted that similar analysis was undertaken after each of days 1 and 2 also. These can be split into QC concerning chip-to-chip integrity as compared to the reference set and similarity analysis of technical replicates. For brevity we describe only the low mass range, but results for the medium mass range were similar.

### Chip-to-chip QC

**Figure 1:**
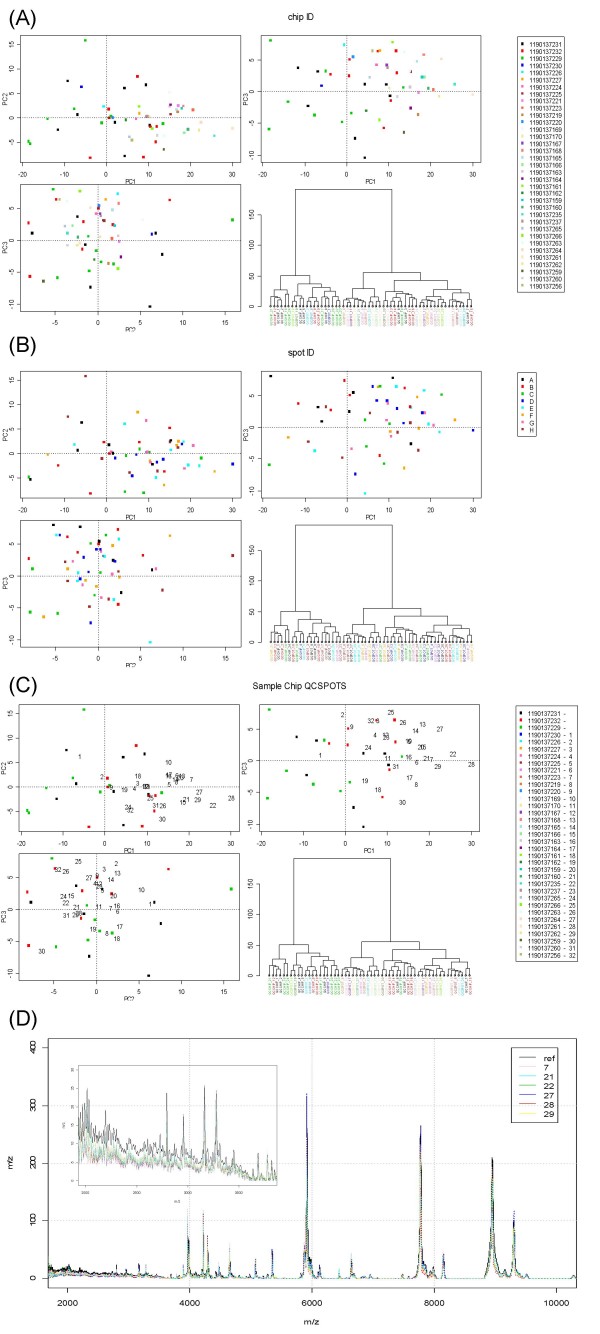
**QC tool output for section of study of patients with different levels of renal function using IMAC-Cu ProteinChips.** Pair-wise plots of the first two principal components (PCs) and a dendrogram showing results of hierarchical clustering using Ward's agglomeration method and the Euclidean distance metric are shown in each panel. Panel (A) shows the plots with the chip ID number indicated by plotting character. The reference set chips are indicated with red, black and green plotting characters and subsequent projections described in the legend. Panel (B) shows the plots with the spot indicated. Panel (C) shows the plots with the reference set indicated by solid plotting characters (red, green and black as in panel (A)) and numbers plotted representing order of QC spectra on sample chips. Panel (D) shows the mean spectra obtained from samples in the reference set and also individual spectra which appear to be on the edge of the PC space in the PC plots in panel (C). The inset plot in panel (D) shows an expanded region of the spectra where the differences from the reference spectra can be observed.

The QC web tool was run selecting no baseline subtraction and default parameters for peak detection and the results page generated. This consists of a table showing the results of the significance test for each QC spot (Table S1 [see Additional file 3]). Figure 1 gives a comprehensive range of displays for assessing spectral quality. The first part of the results page showing pair-wise plots of the first three PCs and the fourth panel a dendrogram showing hierarchical clustering of the peak detected proteomic profiles.

Figure 1(A) shows the chip from which the sample was generated with each colour representing a different chip and results from all QC samples shown with Figure 1(B) showing the analogous results but on a spot basis. By examining these plots any systematic differences in chips and spots can be visualised, indicating the need for closer analysis – in this case there are no obvious patterns and hence no evidence of systematic bias due to either of these variables. The dendrograms in Figures 1(A), (B) and 1(C) are not very enlightening, but they have proved useful in other experiments as the higher level branches often split the samples by day of profile generation. Thus checking the labels and not seeing any relationship with day indicates an experiment where the day effect is small, as in this case. This finding is verified to some extent by the variance components analysis in Section 3.5.

Figure 1(C) shows spectra from the first three chips (i.e. the reference set) indicated by square plotting characters with colours indicating each chip. The projection into the PC space of the subsequent QC spectra from sample chips are indicated by numeric plotting characters (corresponding to the first column of the table which accompany it: Table S1 [see Additional file 3]). By examining this plot and the spectra which are unusual compared to the reference set, i.e. those which are far from the origin (indicated by the intersection of the dotted lines) the progress of the sample run can be monitored. The plot indicates that QC spots 28, 22, 27, 29, 21 and 7 and therefore the spectra which are generated on the same chip should be examined further, but not necessarily rejected immediately. These spectra can be seen plotted on the same axis as the mean spectrum from the reference set in Figure 1(D). It is clear that there are minor aberrant regions, for instance in the regions between 2 and 4 kDa which are shown in the exploded version of the plot where QC spectra from sample chips are generally of lesser intensity than the mean spectra from the reference set. Here the differences are only slight and warrant only a cursory check of the spectra on those chips.

Table S1 provides an additional quantitative method of evaluating QC spectra in comparison to the reference set. It was calculated that 8 PCs make up 90% of the variance in the original data and the significance test was undertaken to see if the MD of these projections of QC spectra from the origin of the PC space was significantly different from zero. As a rule of thumb a cross is placed in the final column of the table if the *p*-value is less than 0.1. This indicates that this spectrum and those on the chip it was generated from should be subjected to further examination. In the web-tool, clicking on the chip ID in the table results in the specific spectrum being plotted in a new window overlaying a realisation of the mean spectra from the reference set. This allows instant visualisation of the spectra and quick investigation of the reason for QC failure.

### QC replicate analysis

**Figure 2:**
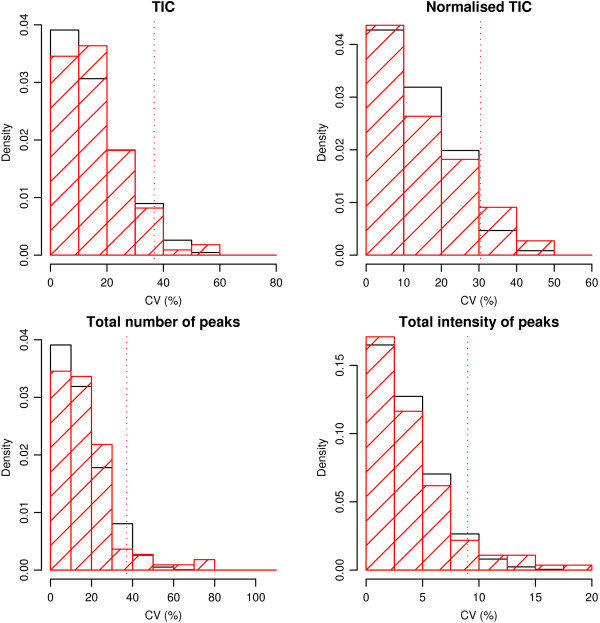
**The black solid bars indicate histograms of coefficient of variations expressed as percentages (CVs) for all possible pairs of QC spectra for the variables of interest in the 2–4 kDa mass region (clockwise from top left, TIC, normalised TIC, total intensity of peaks and total number of peaks).** The dotted line perpendicular to the abscissa indicates the critical value for these empirical significance tests based on the 95% quantile of these measures. The red hatched histograms shows the distribution of CVs calculated for the duplicate technical replicates. CVs which are greater than the critical value will be rejected at the 5% level. These QC fails will be indicated by crosses in Table 1.

The final part of integrated QC analysis is the analysis of replicates. In this case the mass region was split into four equally sized segments for examination of the derived QC parameters. The output of the analysis takes the form of a table, 5 columns of which are devoted to each mass segment. A few selected rows of this output are shown in Table 1 (the full table is shown in Table S2 [see Additional file 4]). A cross or a value greater than zero indicates a discrepancy in this mass segment and this parameter for this pair of spectra as compared to what would be expected from the reference set of spectra. The meaning of the crosses in the first four columns is enhanced by examining Figure 2. These histograms show the null distribution (in black) and the calculated statistics (in red) for each variable, with any calculated statistic greater than the red dotted vertical line being marked with a cross in Table 1 and Table S2.

**Table 1:** Abridged results of replicate analysis presented in web QC tool.

	**2–4 kDa**					**4–6 kDa**					**6–8 kDa**					**8–10 kDa**				
**Sample ID**	**TIC**	**Nm TIC**	**# pk**	**sum pk ints**	**% pk dif**	**TIC**	**Nm TIC**	**# pk**	**sum pk ints**	**% pk dif**	**TIC**	**Nm TIC**	**# pk**	**sum pk ints**	**% pk dif**	**TIC**	**Nm TIC**	**# pk**	**sum pk ints**	**% pk dif**

3965	✓	✓	✓	✓	0	✓	✓	✓	✓	0	✓	✓	✓	✓	0	✓	✓	✓	✓	0
4000	✓	✓	✓	✓	39	✓	✓	✓	✓	3	✓	✓	✓	✓	13	✓	✓	✓	✓	33
4008	✗	✓	✗	✗	16	✗	✓	✗	✓	22	✓	✓	✗	✗	19	✗	✓	✗	✓	9
4021	✓	✓	✓	✓	21	✓	✓	✓	✓	0	✓	✓	✓	✓	0	✓	✓	✓	✓	0
4344	✓	✓	✓	✗	6	✗	✗	✗	✗	0	✗	✗	✗	✗	0	✗	✗	✗	✓	0

For each biological replicate (labelled by sample ID) the technical replicates are compared for various parameters in mass segments of the low mass range (2–4, 4–6, 6–8, 8–10 kDa out of full 2–10 kDa range). The first four of these parameters are the total ion current, the normalised total ion current, the total number of peaks (common and non-common peaks) and the total intensity of peaks. A tick in the columns for each of these parameters indicates that the coefficient of variation (CV) for that variable for these technical replicates is less than the 95% quantile of CVs calculated from all possible pairs of the QC samples. Conversely a cross indicates that they are greater than this empirical limit. The fifth parameter is presented numerically and is the percentage of peaks significantly different in the technical replicates based on an empirical significance test (derived similarly from QC samples). Results for all biological replicates are given in Table S2 [see Additional file 4].

**Figure 3:**
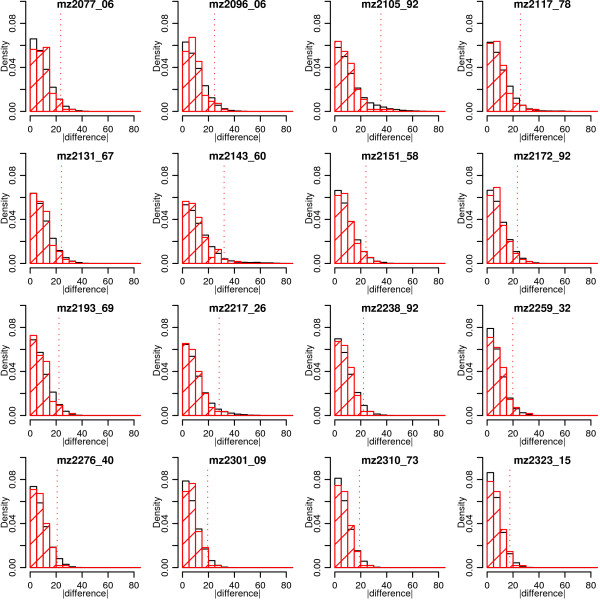
**Demonstration of absolute difference in technical replicate peaks empirical significance methods for 16 conveniently chosen peaks in the 2–4 kDa mass segment.** Solid black histograms show the reference distribution in black with the actual differences indicated by the red hatched histogram bars. Values greater than the critical value (indicated by red dotted line from the abscissa) result in a significant difference in a pair of technical replicates being declared. The percentage of peaks declared significantly different out of the total number of peaks in that mass segment is shown in the fifth column of each mass segment in Table 1.

The figures in the 5^th ^column for each mass segment in Table 1 show the percentage of common peaks in that region for which the absolute difference between the duplicate pair is significantly different from zero. This is also based on empirical significance tests derived from the reference set of QC spectra. Figure 3 shows histograms similar to Figure 2 for 16 conveniently chosen peaks from the 2–4 kDa region. As for the other QC variables, a value greater than the critical value means declaring a significant absolute difference between duplicate technical replicates for a particular peak. To summarize these results, the percentage of peaks declared significantly different out of the total number of peaks in that mass segment is shown in each fifth column in Table 1.

**Figure 4:**
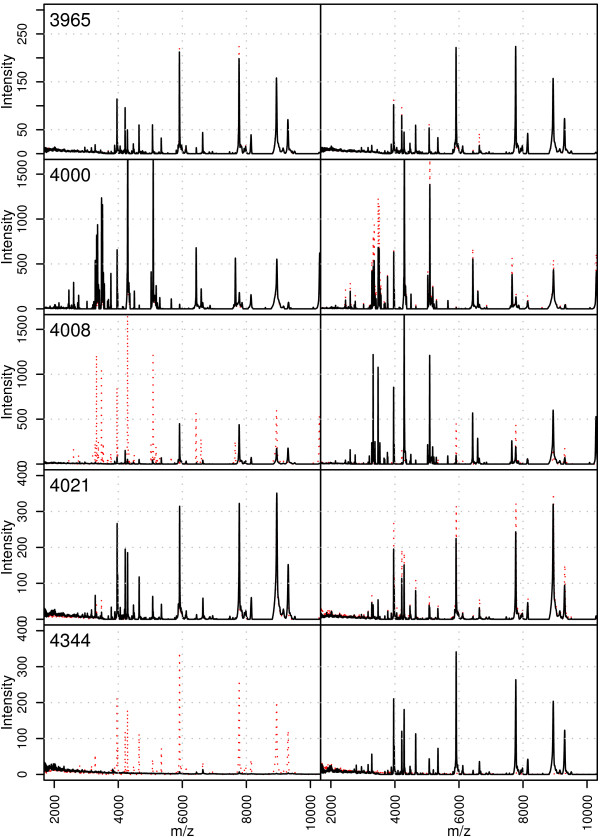
**Duplicate proteomic profiles (technical replicates) of 5 biological samples in the study.** In the left hand panel the first technical replicate is shown with the black solid line and the second technical replicate with the red dotted line and in the right hand panel the second technical replicate is shown with the black solid line and the first technical replicate with the red dotted line. The axes are equivalent in each plot and the mass axes apply to all plots.

Upon observing a cross or a high percentage the appropriate spectra should be examined and a decision made as to whether a further technical replicate should be produced. A few examples corresponding to Table 1 are shown in Figure 4. Here 5 duplicate spectra are shown both overlaid (on the same pair of axes) and adjacent to each other horizontally on separate axes. This is an example of the functionality afforded by the web tool which allows visual examination of duplicate spectra in a number of ways. This allows any spectra to be carefully and thoroughly examined.

The five example duplicate pairs of technical replicates in Figure 4 show a full range of situations identified by the QC tool. Sample 3965 is an example of a biological replicate with two very good technical replicates. In this case no parameters were flagged in any of the mass segments and also none of the peaks in any of the mass segments are declared as being significantly different. The final row of Figure 4 shows the other extreme with the first technical replicate of sample 4344 having no high intensity peaks and only some sinusoidal noise in the region 2–4 kDa whereas the second technical replicate produced a full spectrum. The row of Table 1 representing sample 4344 directly shows what can be seen in the plot of the spectra. In the mass segment between 2 and 4 kDa where there are few peaks, the TIC, normalised TIC and total number of peaks are not flagged as being different, but a problem is identified by the cross in the column indicating the total intensity of peaks and the 6% of peaks which are significantly different between the two spectra. The other three mass segments showed the problem more immediately with red crosses indicating problems in all of these segments which can be clearly seen in the final row of Figure 1. The malfunction in the replicate showing no spectrum was attributed to chip surface malfunction. The second and fourth rows of Figure 4, representing technical replicates for samples 4000 and 4021, show more subtle differences identified by the QC tool. Sample 4000 is not flagged by any of the QC parameters in any of the four mass segments. Instead it is the percentage of peaks which are significantly different that are flagged in 3 out of 4 mass segments. This is quite a common phenomenon which we believe is due to the difference in technical replicates being localised in very sharp peaks in the mass spectra which have a very minor area (in an integral sense) and hence not a large effect on the TIC or other derived QC variables. It is in this situation that the variable measuring the percentage of different peaks is most valuable. Sample 4021 shows a similar phenomenon, but localised only in the first mass segment. Sample 4008 shows a combination of these effects and is hence identified by both failure in the 4 main derived parameters and also in the percentage of peaks which are significantly different between the duplicate samples.

### Final results

After full consideration of QC results 23 samples were selected as requiring a further technical replicate which were generated on 4 IMAC-Cu chips with a QC spot on each chip, as per standard protocol. The new samples were then compared with the identified poor pair of technical replicates and the best pair of these three technical replicates selected for use in subsequent analysis.

### A further example

**Figure 5:**
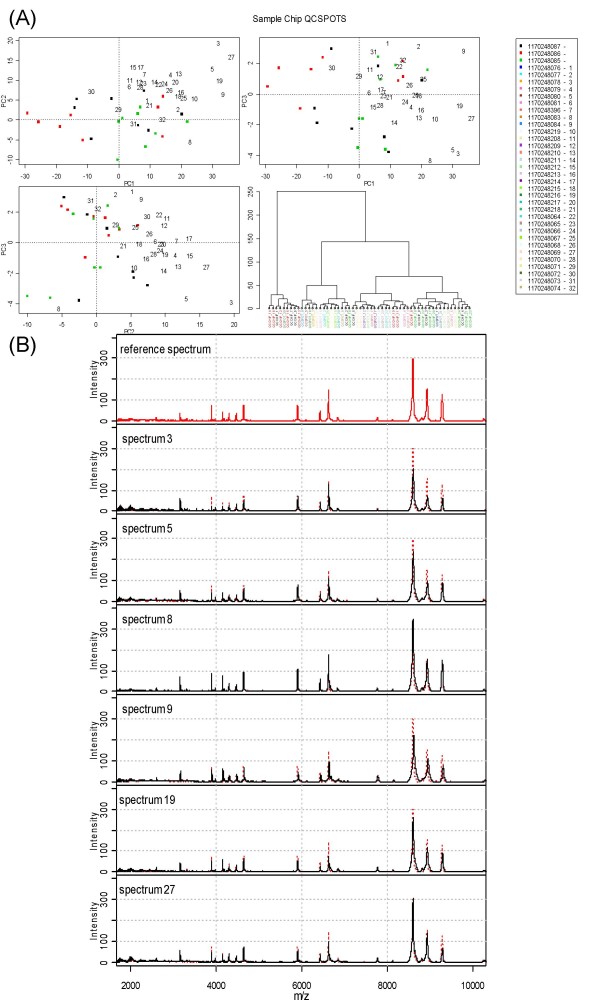
**Selected QC tool output for section of study of patients with different levels of renal function using CM10 ProteinChips.** Panel (A) is similar to Figure 1 panel (C). Panel (B) shows the mean spectra obtained from samples in the reference set and also individual spectra which appear to be on the edge of the PC space in the PC plots in panel (A).

Figure 5 shows results for the CM10 ProteinChip part of the study of renal function. These plots are similar to those in Figure 1 which were described in Section 3.1 in that they are the results of the chip-to-chip QC. The equivalents figures for these results as Figure 1(A) and Figure 1(B) fail to show any systematic bias according to chip and spot. However, Figure 4(A) is different to the previously described experiment (c.f. Figure 1(C)) in that there are a number of projections of spectra which can be found on the edge of the three dimensional PCs space denoted by this panel of plots. In particular the QC spectra from chips 3, 5, 8, 9, 19 and 27 appear to be on the extremes of the space in all three plots. Additionally, these plots were shown to have Mahalanobis distances significantly different from zero in 6 dimensional PC space (*p *< 0.05, data not shown). In Figure 5(B) each of these spectra is shown along with the mean spectra formed by taking the mean over all data points from the spectra which make up the reference set (referred to in the legend as the reference spectrum). Spectra 3 and 5 quite clearly show a decrease in intensity to that observed in the reference spectrum in some of the larger peaks in conjunction with an increase in sinusoidal noise in the region between 2 and 4 kDa, making it apparent why these have been flagged by QC. Spectra 8, 9 and 19 are interesting in that they show a possibility of a drift in the calibration of the machine, evident in the larger peaks between 8 and 10 kDa. In spectra 9 and 19 this is exacerbated by a drop in the intensity in the 6–7 kDa region. Spectrum 27 similarly has a decreased intensity in some peaks, but not in the same manner as those in the other examples shown here. This demonstrates the flexibility of the PCA approach to QC identifying many different kinds of aberrant behaviour in the spectra through one device.

### Variation attributable to measured factors

**Figure 6:**
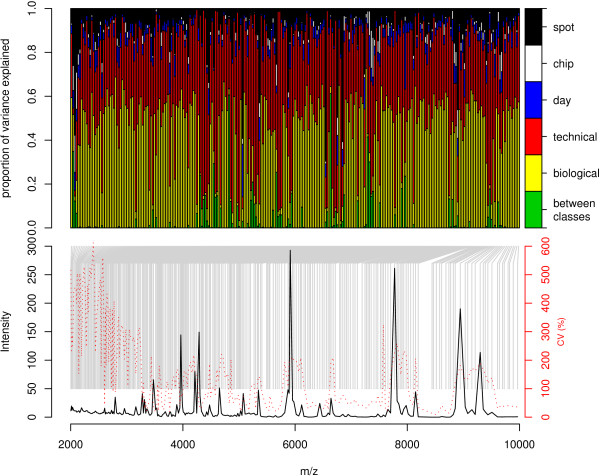
**Variance components, mean and CV spectra for proteomic profiling study of renal function using IMAC-Cu chips).** Each bar in the top panel represents the proportion of variance that can be attributed to biological (within and between classes) and technical (within and between days) components of variation for each peak. The peak corresponding to each bar is denoted by a gray line from the mean/CV spectra in the lower panel.

With high quality data, it is possible to estimate the magnitude of the components of variation which can be attributed to different factors in the design of this experiment. The classical estimation method was used to estimate simple variation components attributing variation to technical and biological components and these results compared with a similar model estimated in OpenBUGS and found to produce similar results. The more complex model with terms for day, chip and spot within the technical variation was fitted and convergence assessed for each parameter for each peak using the scale reduction factor. Figure 6 shows graphically the magnitude of variance components as a proportion of the total variation. This is presented in conjunction with a representation of the mean and CV spectra for intensity allowing the attribution of portions of the variation to each of these factors. Further details regarding the mean and CV spectra are provided by the summary statistics shown in Table S3 [see Additional file 5]. Figure 6 in conjunction with Table 2 show summaries of the results of the variance components analysis in the case where we attribute variance to biological variation (both between groups and within groups) and technical variation (attributed to day, chip, spot and within day). Figure 6 shows that in most cases around half of the variation can be attributed to technical variation (black, white, blue and red bars) and half of the variation to biological variation (green and yellow bars). When summarizing variance components as percentages of the total variance, the median variance in peak intensity can be characterised as consisting of approximately 49% technical variance and 51% biological variance. The median biological component of variance is made up of 0.06% of variation between classes (although it is clear that this is much higher in some cases). Similarly, the technical variation can be split into 4% between day, 1% attributable to chip and 7% attributable to spot with the remaining 37% attributable to within-day variation which cannot be characterised by this analysis. Of the factors in technical variation that could be decomposed it can be seen that the variance due to day of profile determination and the variances due to chip and spot for the mean peak are very small when compared to the unexplained factors in technical variation (Table 2) which could be due to robot performance, laser or detector stability or other factors in the experimental process.

**Table 2:** Summary statistics for variance components of peaks in renal transplant study estimated using crossed analysis of variance.

	**between class**	**biological**	**other technical**	**day**	**chip**	**spot**
Minimum	<0.01	0.99	2.53	0.07	0.02	0.17
1st Quartile	0.02	26.16	30.93	2.22	0.24	4.16
Median	0.06	50.96	34.19	3.57	0.72	6.88
Mean	3.02	41.22	39.72	4.32	1.92	9.80
3rd Quartile	1.09	56.61	39.36	5.51	2.35	11.52
Maximum	61.20	71.30	91.03	30.98	18.11	69.57

Variance components are summarised as percentages of the total variance. Therefore the median peak can be characterised as consisting of approximately 49% technical variance (approximately 3.5% day of profile generation, 1% chip the replicate was generated from, 7% spot the replicate was generated from and 55% other sources) and 51% biological variance (approximately 50.95% within biological classes and 0.05% differences between them).

Comparing these results with those found in other studies provides insights into the comparability of our laboratory with others reported in the literature. For instance, Oberg and colleagues [48] have shown the effectiveness of such techniques in better understanding sources of variation in MS using the iTRAQ relative labelling protocol, de Noo and colleagues [33] have investigated various sample handling steps (storage temperature prior to centrifugation, freeze-thawing and circadian rhythm) and Coombes and colleagues [34] have estimated the proportion of variation due to day and chip (and other technical variation which they refer to as spot-to-spot). They suggest 26% of variation in the profile in due to day-to-day effect and 5% to chip-to-chip effect, while de Noo and colleagues calculate a CV of around 20–30% for day-to-day. The results in terms of percentages for Coombes study are larger than those for the third quartile of all peaks for day and chip in our study (from Table 2 – approximately 7.6% and 3.5%). Furthermore, if three quarters of the peaks found had less than 7.6% of the variation attributable to day and we take this 7.6% and apply it to the third quartile of the CV distribution (from Table S3 [see Additional file 5]) then that indicates approximately a CV of 18% for day, with a number of CVs smaller than this. So at least three-quarters of peaks have a day-to-day CV less than the values obtained in de Noo's investigation.

To our knowledge there has not as yet been an investigation which could demonstrate the proportion of variation due to chip and spot and the fact that the combined effect is smaller than that for day is encouraging. This type of analysis where we investigate experimental bias due to explainable factors is very useful in directly defining potential confounding factors [49]. Hopefully such new knowledge will be useful in experimental design and will result in generally more robust study designs and subsequently better interpretation of study results. In the data analysed here the unexplained technical variation is undoubtedly too large. However, this is a well controlled experiment with rigorous QC. Hence what is actually being demonstrated is the small amount of variation in the proteome which is being examined in studies of un-fractionated serum using the SELDI instrument.

## Conclusion

In this article a novel system for undertaking QC in proteomic profiling studies using MS is presented. This system is multi-facetted and integrated with the database which stores data from the MS instrument and is easily run using a web interface. The multi-facetted nature refers to the functionality of the QC system to monitor the experiment as it progresses and also to evaluate the similarity of duplicates for inclusion/exclusion in subsequent analysis. This is achieved through a convenient web interface available on any networked computer in our laboratory. Additionally, a stand alone GUI is under development which will allow the import of data from other MS instruments.

Rigorous QC has been shown to be an important requirement in proteomic profiling experiments [50]. Although a number of investigations have alluded to some level of QC in their investigation, this often only takes the form of analysis of some larger peaks in terms of CVs of intensities [25,29,35,51-53]. Some investigations have used the calibration sample to perform QC on the intensity axis as well as the mass axis [17]. Again, this is not really adequate because it does not reflect well the true situation in a proteomic profiling experiment where a large number of molecules in a complex mixture are being examined simultaneously rather than only a few pure proteins. The method described here is a step forward in that it is a far more holistic approach to QC which simultaneously takes into account intensities throughout the proteomic profile. This is the advantage of using PCA to perform QC as has been demonstrated in Section 3.1 – a number of different types of aberrant behaviour can be summarized through a small number of composite variables. It can be seen in Figures 1 and 4 that the first PC has a larger spread than the second. This is not uncommon as it is a common occurrence that the first PC is often related to the scale of an object [39] (in this case the sum of intensities) even when working with scale-free transformed data and is apparent here. This could be considered to be a limitation to this method if only the first few PCs are used for QC. However, this is not a concern here as we combine the visual method of plotting the first 3 PCs with a significance test based on the MD. This test allows evaluation over more than just a few PCs so what might not be seen in plots will be taken into account in the significance test.

The chip-to-chip QC procedure was inspired by the excellent work described by Coombes and colleagues [34], but with some important modifications. Their basic strategy was to define the "base profile" (another term for the reference set) derived from QC samples (pooled samples of the sample type under consideration) on four ProteinChips run over a number of days. This base profile is then examined and peak clusters are identified using a simple peak finding algorithm. PCA was then used and the first 7 PCs chosen as the composite variables which define this base profile. The QC sample was spotted onto 2 random spots on each sample ProteinChip and then the profiles matched to the peaks detected in the base profile, and these matched peaks projected into the PC space. The MD between these two QC profiles and the origin of the QC space for each sample chip is then calculated and this value compared with zero using a statistical significance test. If there is evidence that the distance between QC profiles and the origin from both chips is significantly different from zero then the chip is rejected by their QC, i.e. with one failure QC is passed. Our modifications to this strategy are simple and justifiable, but make the method preferable in a number of ways. Firstly, the QC samples are not matched to peak clusters obtained from the base profile. Instead the data is peak detected simultaneously each time there is new data to compare with the reference set. This is preferable as it allows the inclusion of features later found in the QC samples that were not present in the reference set, for example, contamination of the chip surface. The second modification is the use of a study-specific number of PCs in the significance test (the number that contain greater than 90% of the variance), rather than the less flexible 7 PCs advocated by Coombes and colleagues. Also, it could be suggested that using a reference set based on profiles from only one day could be making the QC method more stringent as it does not include day-to-day variation. We feel these adjustments make our chip-to-chip QC procedure more flexible and applicable to a wider number of situations (different disease and chip types and samples).

Another important modification in our experiments is that the QC samples are distributed equally on all spots across the chips, whereas in the Coombes study the QC samples were applied to the same spot on all chips. This could introduce some bias into the procedure if there are systematic problems with the spots chosen for the QC sample. Additionally, it has already been noted that the method of Coombes and colleagues is expensive in terms of experimental units with 25% of sample chips devoted to the use of QC [32] (c.f. 12.5% in this study).

Although consistency of the reference set is checked visually at the beginning of a study, it is also possible to update it later by trimming the extremes of the data if more stringent cut-offs are required using an option in the web-tool. If changes in the experiment are thought to be more extreme, then the QC spectra which define the reference set can be changed. The necessity of changes in the reference set can be due to a change in intersession laser performance (as measured through TIC) or changes in laboratory temperature (data not shown). In these situations a leave-*k*-out approach to defining the reference set can be useful, e.g. defining the reference set with QC profiles from other days in the study and not the first three chips. This requires minor manipulation of the sample list and has proved invaluable on some occasions.

In the variance components analysis described previously it has been shown that there is a relatively large amount of technical variation, but a reassuringly small amount of it is due to the day of profile generation, the ProteinChip or the spot on the ProteinChip. Other authors have shown the effect of reasonable reproducibility over sessions [54] and the small, but detrimental, effect it has on classification algorithms and no doubt also class comparisons. This is what would be expected based on this analysis and also that of de Noo and colleagues [33], who recommend performing all experiments on one day or standardly correcting for day-to-day variation.

The requirement of rigorous QC for proteomic profiling by MS are not unique and occur in a number of expression analyses techniques [32], depletion techniques [55], more complex designed experiments [56] and filtering of poor quality spectra in peptide identification sequencing using support vector machines [57]. These are techniques where this QC strategy of PCA and empirical significance testing could also prove effective. In addition to the intra-experiment QC described in this manuscript it is also important to perform inter-experiment QC. This type of QC will monitor the performance of the MS instrument over time and is particularly important prior to the commencement of any proteomic profiling study. Variables similar to those described here such as TIC can be useful in this type of analysis as a coarse indicator of instrument performance using quality control chart techniques based on means of previous inter-experiment QC measures and confidence interval limits.

Good clinical practice (EU directive 2001/20/EC) and good clinical laboratory practice [58] are a requirement of clinical trials which should be adopted in clinical proteomic profiling studies [7] and one of the facets which should be included is QC protocols. Similarly the methods described in this manuscript are suggested to be part of the essential reporting of the clinical proteomic profiling study in a recent review [7]. It is hoped that the combination of standard operating procedures and rigorous QC will allow production of high quality proteomic profiles; such techniques can only improve the quality of MS-based proteomic profiling studies.

## Competing interests

The authors declare that they have no competing interests.

## Authors' contributions

REB identified the need for an automated method of QC and initiated the programme of research together with PJS. DNP, DAC and REB identified measures to be used to monitor the quality of proteomic profiling experiments. DAC and JHB developed the statistical methods of QC. DAC wrote the scripts to undertake QC in the R software. DNP wrote the software to allow integrated QC as a web-tool added to the commercial software for managing the mass spectrometer. AJS produced the experimental data and evaluated the developing web-tool. DT evaluated the QC tool by visually inspecting raw spectral data and evaluating evolving methods of QC. DAC and DNP drafted the manuscript and all authors provided comments and approved the manuscript for submission.

## Availability and requirements

An executable file which can be used to run the QC procedures described in this paper will be available at . This file can be used by anybody who has a Ciphergen MySQL database of results and follows the instructions detailed on the website. Further developments of the QC software will also be posted at this site.

## Supplementary Material

Additional file 1 **Supplementary Figure S1.** This file contains a figure describing the randomisation and quality control scheme layout diagrammatically. Click here for file

Additional file 2 **Pre-processing methods details.** This file contains details of the pre-processing scheme for the mass spectrometry data not included in the main text for brevity. Click here for file

Additional file 3 **Table S1.** This file contains the results of QC spot analysis in a supplementary table. Click here for file

Additional file 4 **Table S2.** This file contains the full results from the QC replicate analysis, i.e. the unabridged version of Table 1. Click here for file

Additional file 5 **Table S3.** This file contains a table of summary statistics concerning the mean spectra and CV spectra displayed in Figure 6. Click here for file
