# Supplementary material for: Integrated multi-level quality control for proteomic profiling studies using mass spectrometry
Source: BMC Bioinformatics. 2008 Dec 4;9:519. doi: 10.1186/1471-2105-9-519 (PMC2657802; doi:10.1186/1471-2105-9-519)
Supplement: Additional file 1 — Supplementary Figure S1. This file contains a figure describing the randomisation and quality control scheme layout diagrammatically. [file 1471-2105-9-519-S1.ppt]

## Slide 1
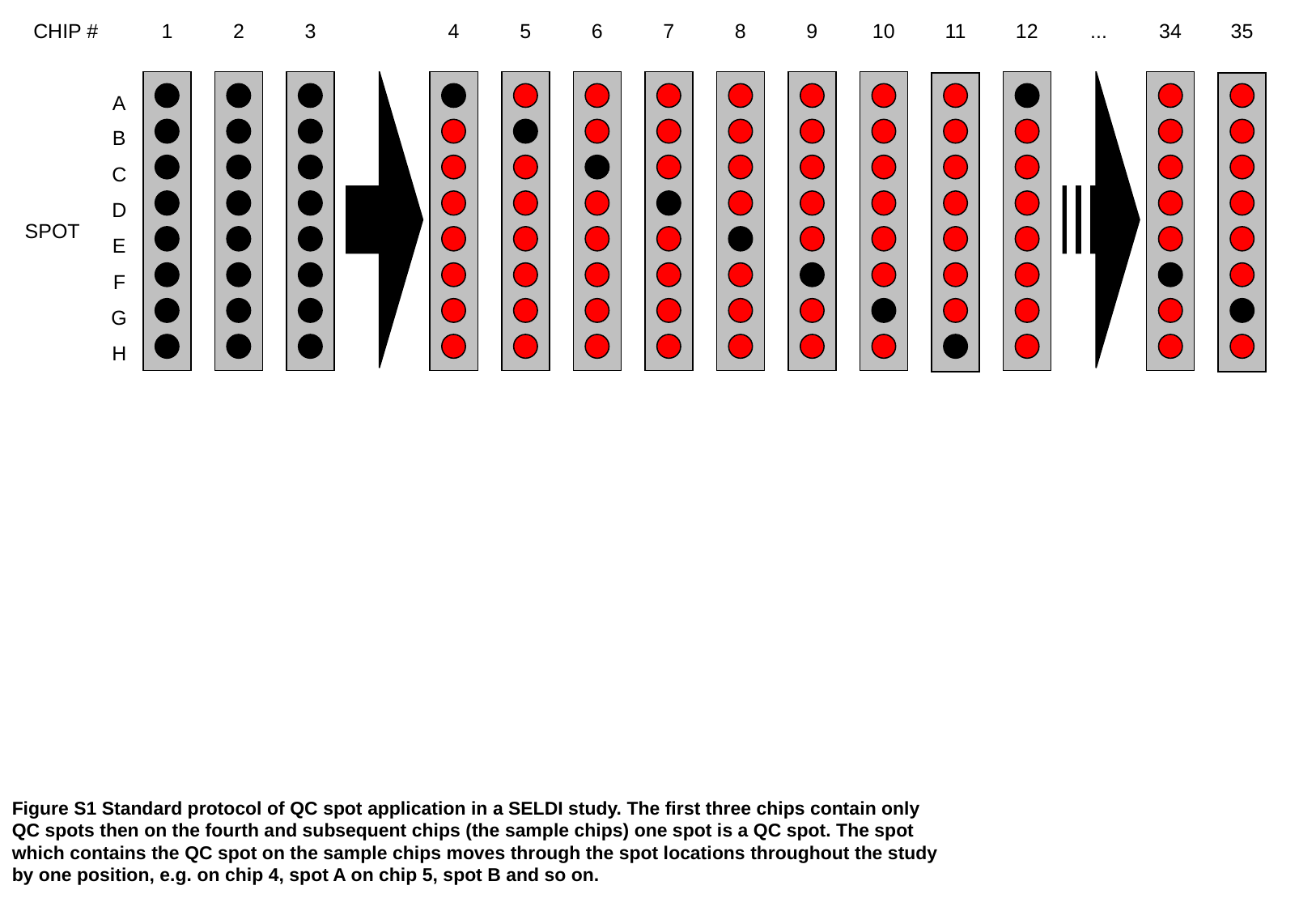

CHIP #
1
2
3
4
5
6
7
8
9
10
11
12
...
34
35
A
B
C
SPOT
D
E
F
G
H
Figure S1 Standard protocol of QC spot application in a SELDI study. The first three chips contain only QC spots then on the fourth and subsequent chips (the sample chips) one spot is a QC spot. The spot which contains the QC spot on the sample chips moves through the spot locations throughout the study by one position, e.g. on chip 4, spot A on chip 5, spot B and so on.
