# Supplementary material for: Integrated multi-level quality control for proteomic profiling studies using mass spectrometry
Source: BMC Bioinformatics. 2008 Dec 4;9:519. doi: 10.1186/1471-2105-9-519 (PMC2657802; doi:10.1186/1471-2105-9-519)
Supplement: Additional file 2 — Pre-processing methods details. This file contains details of the pre-processing scheme for the mass spectrometry data not included in the main text for brevity. [file 1471-2105-9-519-S2.doc]

In section 2.3 of the manuscript, ‘Integrated multi-level quality control for proteomic profiling studies using mass spectrometry’ by Cairns *et al*., a number of pre-processing steps (baseline subtraction, internal normalisation and peak detection) in an pre-analysis pipeline are alluded to, but are not described fully for brevity. These are described in this document for the interested reader.

- Baseline Subtraction

The raw spectra exhibit an elevated baseline, especially at low m/z values, that is mostly the result of chemical and detector noise. It is essential to subtract this noise from each spectrum to produce a more accurate intensity value and in this study a locally weighted regression (LOESS) algorithm was applied. This is a two stage process where initially a baseline was estimated from the unprocessed spectrum. During the second stage, those intensity values that deviated by more than one standard deviation from the initial baseline were temporarily replaced by the baseline value and the smoothing technique was re-applied. The estimated values from this model were then subtracted from the raw data to obtain the baseline subtracted spectra. This is a modified version of the method described by Wagner and colleagues [1].

- Internal normalisation

Normalisation was carried out for each spectrum by dividing each intensity value by the maximum intensity value within that spectrum. The intensity values are therefore represented by the range of numeric values 0 to 1. This allowed for better comparison between spectra in those cases where the number and relative intensities of peaks are similar, but the absolute intensity values differ significantly. However, normalisation between spectra, for example on the basis of total ion current, is avoided as this may disguise real biological differences between samples [2].

- Peak Detection

Peak detection was undertaken to remove noise and to identify conserved peaks among spectra. The method applied is based on that described previously [3, 4] with some minor modifications. There are several steps in the process described algorithmically below. Supposing there are *m* spectra and *k* data points:

- 1. Each spectrum was smoothed using moving averages. More precisely, for each of the *m* spectra, for *n* = 2, 3, …, 20, windows 2*n*+1 points wide (*n* points on either side of each m/z value, from the (*n*+1)th to the (*k*-*n*)th value) were formed, and the means of the 2*n*+1 intensities were calculated. This results in 19 successively smoother versions of each spectrum for each of the original *m* spectra.
  2. Peaks of the smoothed spectra were identified. A peak was defined as a point in the smoothed spectrum which is a local maximum in the sense that there are at least *n* points lower at each side where *n*=2, 3, ..., 20 once again.
  3. For each m/z value the number of times a peak was detected was counted by considering all the smoothed spectra for each sample. The theoretical maximum number of times a peak could occur is thus *m*×19 (if the peak is apparent at that m/z value in each spectrum and at each resolution). At each m/z point a peak was counted if it lies within a tolerance of 0.1%; hence some peaks will be counted in a number of m/z bins.
  4. Centres of peak clusters were identified. The peak frequency distribution produced in the previous step was smoothed using a simple 7-point moving average. Centres of peak clusters were identified as local maxima in this distribution, in the sense that the numbers of peaks two points either side are monotonically decreasing or equal. This results in a final “master” list of peaks, typically in the order of a few hundred.
  5. For each spectrum, the peak intensity associated with that common peak was determined by selecting the maximum intensity within a window of tolerance of 0.1% of the master peak. This process resulted in a matrix of intensity measurements for each profile at each of the master peaks identified in step 4.

Steps 1 through 4 are further described by Barrett and Cairns [4] with diagrams for further explanation.

# References

1. Wagner M, Naik D, Pothen A: **Protocols for disease classification from mass spectrometry data**. *Proteomics* 2003, **3**(9):1692-1698.

2. Cairns DA, Thompson D, Perkins DN, Stanley AJ, Selby PJ, Banks RE: **Proteomic profiling using mass spectrometry - does normalising by total ion current potentially mask some biological differences?** *Proteomics* 2008, **8**(1):21-27.

3. Rogers MA, Clarke P, Noble J, Munro NP, Paul A, Selby PJ, Banks RE: **Proteomic profiling of urinary proteins in renal cancer by surface enhanced laser desorption ionization and neural-network analysis: Identification of key issues affecting potential clinical utility**. *Cancer Research* 2003, **63**(20):6971-6983.

4. Barrett JH, Cairns DA: **Application of the random forest classification method to peaks detected from mass spectrometric proteomic profiles of cancer patients and controls**. *Stat Appl Genet Mol Biol* 2008, **7**:Article4.
