# Supplementary material for: Integrated multi-level quality control for proteomic profiling studies using mass spectrometry
Source: BMC Bioinformatics. 2008 Dec 4;9:519. doi: 10.1186/1471-2105-9-519 (PMC2657802; doi:10.1186/1471-2105-9-519)
Supplement: Additional file 3 — Table S1. This file contains the results of QC spot analysis in a supplementary table. [file 1471-2105-9-519-S3.doc]

Table S1 Results of QC for renal function study with IMAC30 chips. Chip # refers to the chip position in the full sample run and Chip ID the unique chip identifier. The MD is the Mahalanobis distance of the projection of that QC profile in PC space and the *p*-value refers to a significance test of whether this difference is significantly different to zero.

| **Chip #** | **Chip ID** | **MD** | ***p*-value** |  |
| --- | --- | --- | --- | --- |
| 1 | 1190137230 | 2.59 | 0.957 | 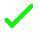 |
| 2 | 1190137226 | 3.72 | 0.881 | 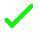 |
| 3 | 1190137227 | 4.61 | 0.798 | 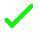 |
| 4 | 1190137224 | 1.44 | 0.993 | 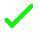 |
| 5 | 1190137225 | 4.22 | 0.837 | 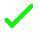 |
| 6 | 1190137221 | 3.55 | 0.895 | 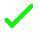 |
| 7 | 1190137223 | 5.07 | 0.750 | 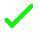 |
| 8 | 1190137219 | 6.85 | 0.553 | 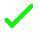 |
| 9 | 1190137220 | 4.65 | 0.795 | 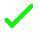 |
| 10 | 1190137169 | 5.83 | 0.666 | 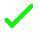 |
| 11 | 1190137170 | 3.28 | 0.916 | 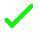 |
| 12 | 1190137167 | 3.92 | 0.864 | 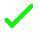 |
| 13 | 1190137168 | 8.80 | 0.359 | 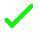 |
| 14 | 1190137165 | 5.03 | 0.754 | 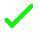 |
| 15 | 1190137166 | 7.06 | 0.530 | 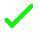 |
| 16 | 1190137163 | 3.76 | 0.878 | 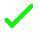 |
| 17 | 1190137164 | 6.01 | 0.646 | 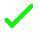 |
| 18 | 1190137161 | 5.83 | 0.666 | 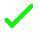 |
| 19 | 1190137162 | 5.44 | 0.710 | 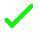 |
| 20 | 1190137159 | 4.70 | 0.789 | 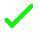 |
| 21 | 1190137160 | 6.10 | 0.636 | 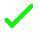 |
| 22 | 1190137235 | 8.10 | 0.424 | 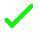 |
| 23 | 1190137237 | 2.47 | 0.963 | 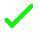 |
| 24 | 1190137265 | 5.50 | 0.703 | 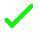 |
| 25 | 1190137266 | 4.97 | 0.761 | 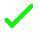 |
| 26 | 1190137263 | 5.86 | 0.663 | 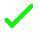 |
| 27 | 1190137264 | 9.32 | 0.316 | 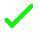 |
| 28 | 1190137261 | 10.64 | 0.223 | 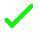 |
| 29 | 1190137262 | 6.80 | 0.558 | 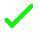 |
| 30 | 1190137259 | 12.82 | 0.118 | 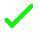 |
| 31 | 1190137260 | 4.58 | 0.801 | 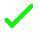 |
| 32 | 1190137256 | 3.38 | 0.909 | 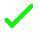 |

As we can see there were no spectra with *p*-values below 0.1 in this case. However, some of the smallest p-values are those observed as unusual in the PCA plots, e.g. 27 and 28. Table S1 also shows additional samples close to being flagged such as sample 30. This is most likely due to variation in one of the PCs not plotted in Figure 1(C), as although PC3 versus PC2 shows sample 30 somewhat isolated – it does not appear to be enough to warrant such a small *p*-value.
