# Supplementary material for: Integrated multi-level quality control for proteomic profiling studies using mass spectrometry
Source: BMC Bioinformatics. 2008 Dec 4;9:519. doi: 10.1186/1471-2105-9-519 (PMC2657802; doi:10.1186/1471-2105-9-519)
Supplement: Additional file 4 — Table S2. This file contains the full results from the QC replicate analysis, i.e. the unabridged version of Table 1. [file 1471-2105-9-519-S4.doc]

Table S2 Results of replicate analysis presented in web QC tool. For each biological replicate (labelled by sample ID) the technical replicates are compared for various parameters in mass segments of the low mass range (2-4, 4-6, 6-8, 8-10kDa out of full 2-10kDa range). The first four of these parameters are the total ion current, the normalised total ion current, the total number of peaks (common and non-common peaks) and the total intensity of peaks. A tick in the columns for each of these parameters indicates that the coefficient of variation (CV) for that variable for these technical replicates are less than the 95% quantile of CVs calculated from all possible pairs of the QC samples. Conversely a cross indicates that they are greater than this empirical limit. The fifth parameter is presented numerically and is the percentage of peaks significantly different in the technical replicate based on an empirical significance test (derived similarly from QC samples).

| Sample ID | 2-4kDa | | | | | 4-6kDa | | | | | 6-8kDa | | | | | 8-10kDa | | | | |
| --- | --- | --- | --- | --- | --- | --- | --- | --- | --- | --- | --- | --- | --- | --- | --- | --- | --- | --- | --- | --- |
|  | TIC | Normalised TIC | Total number of peaks | Total intensity of peaks | %age of peaks different | TIC | Normalised TIC | Total number of peaks | Total intensity of peaks | %age of peaks different | TIC | Normalised TIC | Total number of peaks | Total intensity of peaks | %age of peaks different | TIC | Normalised TIC | Total number of peaks | Total intensity of peaks | %age of peaks different |
| 3862 | 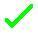 | 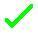 | 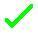 | 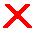 | 0 | 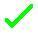 | 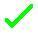 | 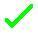 | 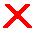 | 0 | 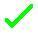 | 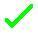 | 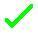 | 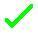 | 0 | 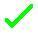 | 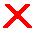 | 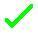 | 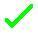 | 0 |
| 3863 | 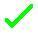 | 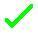 | 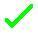 | 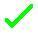 | 0 | 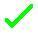 | 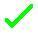 | 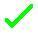 | 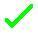 | 0 | 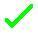 | 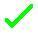 | 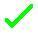 | 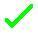 | 0 | 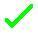 | 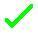 | 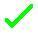 | 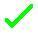 | 0 |
| 3927 | 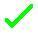 | 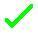 | 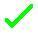 | 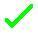 | 0 | 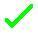 | 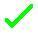 | 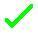 | 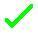 | 0 | 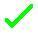 | 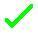 | 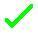 | 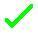 | 0 | 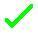 | 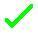 | 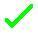 | 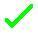 | 0 |
| 3937 | 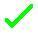 | 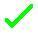 | 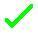 | 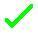 | 0 | 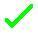 | 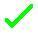 | 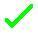 | 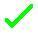 | 0 | 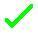 | 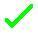 | 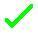 | 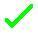 | 0 | 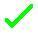 | 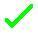 | 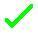 | 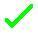 | 0 |
| 3941 | 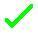 | 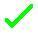 | 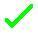 | 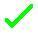 | 1 | 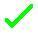 | 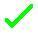 | 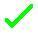 | 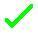 | 0 | 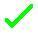 | 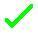 | 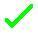 | 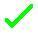 | 0 | 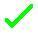 | 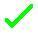 | 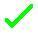 | 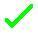 | 0 |
| 3947 | 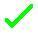 | 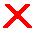 | 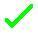 | 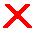 | 0 | 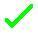 | 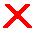 | 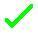 | 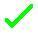 | 0 | 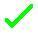 | 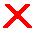 | 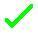 | 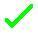 | 0 | 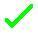 | 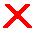 | 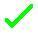 | 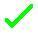 | 0 |
| 3949 | 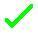 | 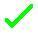 | 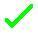 | 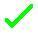 | 0 |  |  |  |  | 0 |  |  |  |  | 0 |  |  |  |  | 0 |
| 3964 |  |  |  |  | 0 |  |  |  |  | 0 |  |  |  |  | 0 |  |  |  |  | 0 |
| 3965 |  |  |  |  | 0 |  |  |  |  | 0 |  |  |  |  | 0 |  |  |  |  | 0 |
| 3966 |  |  |  |  | 0 |  |  |  |  | 0 |  |  |  |  | 0 |  |  |  |  | 0 |
| 3976 |  |  |  |  | 0 |  |  |  |  | 0 |  |  |  |  | 0 |  |  |  |  | 0 |
| 3998 |  |  |  |  | 0 |  |  |  |  | 0 |  |  |  |  | 0 |  |  |  |  | 0 |
| 4000 |  |  |  |  | 39 |  |  |  |  | 3 |  |  |  |  | 13 |  |  |  |  | 33 |
| 4001 |  |  |  |  | 0 |  |  |  |  | 0 |  |  |  |  | 2 |  |  |  |  | 0 |
| 4007 |  |  |  |  | 3 |  |  |  |  | 12 |  |  |  |  | 6 |  |  |  |  | 0 |
| 4008 |  |  |  |  | 16 |  |  |  |  | 22 |  |  |  |  | 19 |  |  |  |  | 9 |
| 4011 |  |  |  |  | 0 |  |  |  |  | 0 |  |  |  |  | 0 |  |  |  |  | 0 |
| 4012 |  |  |  |  | 0 |  |  |  |  | 0 |  |  |  |  | 0 |  |  |  |  | 0 |
| 4021 |  |  |  |  | 21 |  |  |  |  | 0 |  |  |  |  | 0 |  |  |  |  | 0 |
| 4022 |  |  |  |  | 7 |  |  |  |  | 0 |  |  |  |  | 0 |  |  |  |  | 0 |
| 4033 |  |  |  |  | 0 |  |  |  |  | 0 |  |  |  |  | 0 |  |  |  |  | 0 |
| 4039 |  |  |  |  | 1 |  |  |  |  | 3 |  |  |  |  | 4 |  |  |  |  | 0 |
| 4047 |  |  |  |  | 0 |  |  |  |  | 0 |  |  |  |  | 0 |  |  |  |  | 0 |
| 4051 |  |  |  |  | 0 |  |  |  |  | 0 |  |  |  |  | 0 |  |  |  |  | 0 |
| 4065 |  |  |  |  | 0 |  |  |  |  | 0 |  |  |  |  | 0 |  |  |  |  | 0 |
| 4079 |  |  |  |  | 0 |  |  |  |  | 0 |  |  |  |  | 0 |  |  |  |  | 0 |
| 4084 |  |  |  |  | 0 |  |  |  |  | 0 |  |  |  |  | 0 |  |  |  |  | 0 |
| 4089 |  |  |  |  | 0 |  |  |  |  | 0 |  |  |  |  | 0 |  |  |  |  | 0 |
| 4100 |  |  |  |  | 0 |  |  |  |  | 0 |  |  |  |  | 0 |  |  |  |  | 0 |
| 4109 |  |  |  |  | 0 |  |  |  |  | 0 |  |  |  |  | 0 |  |  |  |  | 0 |
| 4111 |  |  |  |  | 6 |  |  |  |  | 0 |  |  |  |  | 2 |  |  |  |  | 6 |
| 4118 |  |  |  |  | 1 |  |  |  |  | 1 |  |  |  |  | 6 |  |  |  |  | 0 |
| 4122 |  |  |  |  | 8 |  |  |  |  | 1 |  |  |  |  | 0 |  |  |  |  | 0 |
| 4133 |  |  |  |  | 0 |  |  |  |  | 0 |  |  |  |  | 0 |  |  |  |  | 0 |
| 4145 |  |  |  |  | 0 |  |  |  |  | 1 |  |  |  |  | 0 |  |  |  |  | 0 |
| 4154 |  |  |  |  | 0 |  |  |  |  | 0 |  |  |  |  | 0 |  |  |  |  | 0 |
| 4161 |  |  |  |  | 0 |  |  |  |  | 1 |  |  |  |  | 0 |  |  |  |  | 0 |
| 4164 |  |  |  |  | 0 |  |  |  |  | 0 |  |  |  |  | 0 |  |  |  |  | 0 |
| 4172 |  |  |  |  | 55 |  |  |  |  | 4 |  |  |  |  | 9 |  |  |  |  | 39 |
| 4176 |  |  |  |  | 0 |  |  |  |  | 0 |  |  |  |  | 0 |  |  |  |  | 0 |
| 4192 |  |  |  |  | 0 |  |  |  |  | 0 |  |  |  |  | 0 |  |  |  |  | 0 |
| 4214 |  |  |  |  | 0 |  |  |  |  | 0 |  |  |  |  | 0 |  |  |  |  | 0 |
| 4243 |  |  |  |  | 0 |  |  |  |  | 0 |  |  |  |  | 0 |  |  |  |  | 0 |
| 4268 |  |  |  |  | 2 |  |  |  |  | 7 |  |  |  |  | 0 |  |  |  |  | 3 |
| 4269 |  |  |  |  | 0 |  |  |  |  | 0 |  |  |  |  | 0 |  |  |  |  | 0 |
| 4270 |  |  |  |  | 13 |  |  |  |  | 11 |  |  |  |  | 13 |  |  |  |  | 3 |
| 4272 |  |  |  |  | 0 |  |  |  |  | 0 |  |  |  |  | 0 |  |  |  |  | 0 |
| 4273 |  |  |  |  | 0 |  |  |  |  | 0 |  |  |  |  | 0 |  |  |  |  | 0 |
| 4274 |  |  |  |  | 0 |  |  |  |  | 0 |  |  |  |  | 0 |  |  |  |  | 0 |
| 4275 |  |  |  |  | 0 |  |  |  |  | 0 |  |  |  |  | 0 |  |  |  |  | 0 |
| 4276 |  |  |  |  | 0 |  |  |  |  | 1 |  |  |  |  | 2 |  |  |  |  | 0 |
| 4277 |  |  |  |  | 0 |  |  |  |  | 0 |  |  |  |  | 0 |  |  |  |  | 0 |
| 4300 |  |  |  |  | 0 |  |  |  |  | 0 |  |  |  |  | 0 |  |  |  |  | 0 |
| 4304 |  |  |  |  | 0 |  |  |  |  | 0 |  |  |  |  | 0 |  |  |  |  | 0 |
| 4305 |  |  |  |  | 0 |  |  |  |  | 0 |  |  |  |  | 0 |  |  |  |  | 0 |
| 4344 |  |  |  |  | 6 |  |  |  |  | 0 |  |  |  |  | 0 |  |  |  |  | 0 |
| 4353 |  |  |  |  | 0 |  |  |  |  | 0 |  |  |  |  | 0 |  |  |  |  | 0 |
| 4359 |  |  |  |  | 0 |  |  |  |  | 0 |  |  |  |  | 0 |  |  |  |  | 0 |
| 4362 |  |  |  |  | 0 |  |  |  |  | 0 |  |  |  |  | 0 |  |  |  |  | 0 |
| 4365 |  |  |  |  | 0 |  |  |  |  | 0 |  |  |  |  | 0 |  |  |  |  | 0 |
| 4410 |  |  |  |  | 0 |  |  |  |  | 0 |  |  |  |  | 2 |  |  |  |  | 0 |
| 4411 |  |  |  |  | 0 |  |  |  |  | 1 |  |  |  |  | 0 |  |  |  |  | 0 |
| 4428 |  |  |  |  | 0 |  |  |  |  | 0 |  |  |  |  | 0 |  |  |  |  | 0 |
| 4502 |  |  |  |  | 0 |  |  |  |  | 0 |  |  |  |  | 0 |  |  |  |  | 0 |
| 4509 |  |  |  |  | 0 |  |  |  |  | 0 |  |  |  |  | 0 |  |  |  |  | 0 |
| 4555 |  |  |  |  | 42 |  |  |  |  | 1 |  |  |  |  | 7 |  |  |  |  | 0 |
| 4556 |  |  |  |  | 0 |  |  |  |  | 0 |  |  |  |  | 0 |  |  |  |  | 0 |
| 4567 |  |  |  |  | 0 |  |  |  |  | 3 |  |  |  |  | 0 |  |  |  |  | 0 |
| 4584 |  |  |  |  | 0 |  |  |  |  | 0 |  |  |  |  | 0 |  |  |  |  | 0 |
| 4585 |  |  |  |  | 0 |  |  |  |  | 0 |  |  |  |  | 0 |  |  |  |  | 0 |
| 4586 |  |  |  |  | 0 |  |  |  |  | 0 |  |  |  |  | 0 |  |  |  |  | 0 |
| 4587 |  |  |  |  | 0 |  |  |  |  | 0 |  |  |  |  | 0 |  |  |  |  | 0 |
| 4588 |  |  |  |  | 0 |  |  |  |  | 0 |  |  |  |  | 0 |  |  |  |  | 0 |
| 4590 |  |  |  |  | 0 |  |  |  |  | 0 |  |  |  |  | 0 |  |  |  |  | 0 |
| 4591 |  |  |  |  | 0 |  |  |  |  | 0 |  |  |  |  | 0 |  |  |  |  | 0 |
| 4598 |  |  |  |  | 0 |  |  |  |  | 0 |  |  |  |  | 0 |  |  |  |  | 0 |
| 4637 |  |  |  |  | 0 |  |  |  |  | 0 |  |  |  |  | 0 |  |  |  |  | 0 |
| 4640 |  |  |  |  | 0 |  |  |  |  | 0 |  |  |  |  | 0 |  |  |  |  | 0 |
| 4641 |  |  |  |  | 0 |  |  |  |  | 0 |  |  |  |  | 0 |  |  |  |  | 0 |
| 4662 |  |  |  |  | 0 |  |  |  |  | 0 |  |  |  |  | 0 |  |  |  |  | 0 |
| 5318 |  |  |  |  | 0 |  |  |  |  | 0 |  |  |  |  | 0 |  |  |  |  | 0 |
| 5319 |  |  |  |  | 0 |  |  |  |  | 0 |  |  |  |  | 0 |  |  |  |  | 0 |
| 5320 |  |  |  |  | 18 |  |  |  |  | 0 |  |  |  |  | 0 |  |  |  |  | 0 |
| 5321 |  |  |  |  | 0 |  |  |  |  | 0 |  |  |  |  | 0 |  |  |  |  | 0 |
| 5322 |  |  |  |  | 0 |  |  |  |  | 0 |  |  |  |  | 0 |  |  |  |  | 0 |
| 5323 |  |  |  |  | 0 |  |  |  |  | 0 |  |  |  |  | 0 |  |  |  |  | 0 |
| 5324 |  |  |  |  | 1 |  |  |  |  | 0 |  |  |  |  | 0 |  |  |  |  | 0 |
| 5325 |  |  |  |  | 0 |  |  |  |  | 0 |  |  |  |  | 0 |  |  |  |  | 0 |
| 5327 |  |  |  |  | 0 |  |  |  |  | 0 |  |  |  |  | 0 |  |  |  |  | 0 |
| 5328 |  |  |  |  | 0 |  |  |  |  | 0 |  |  |  |  | 0 |  |  |  |  | 0 |
| 5333 |  |  |  |  | 1 |  |  |  |  | 0 |  |  |  |  | 0 |  |  |  |  | 0 |
| 5335 |  |  |  |  | 0 |  |  |  |  | 0 |  |  |  |  | 0 |  |  |  |  | 0 |
| 5337 |  |  |  |  | 0 |  |  |  |  | 0 |  |  |  |  | 0 |  |  |  |  | 0 |
| 5338 |  |  |  |  | 0 |  |  |  |  | 0 |  |  |  |  | 0 |  |  |  |  | 0 |
| 5345 |  |  |  |  | 0 |  |  |  |  | 0 |  |  |  |  | 0 |  |  |  |  | 0 |
| 5346 |  |  |  |  | 0 |  |  |  |  | 0 |  |  |  |  | 0 |  |  |  |  | 0 |
| 5348 |  |  |  |  | 4 |  |  |  |  | 1 |  |  |  |  | 0 |  |  |  |  | 0 |
| 5349 |  |  |  |  | 0 |  |  |  |  | 0 |  |  |  |  | 0 |  |  |  |  | 0 |
| 5350 |  |  |  |  | 0 |  |  |  |  | 0 |  |  |  |  | 2 |  |  |  |  | 0 |
| 5351 |  |  |  |  | 0 |  |  |  |  | 0 |  |  |  |  | 0 |  |  |  |  | 0 |
| 5352 |  |  |  |  | 0 |  |  |  |  | 0 |  |  |  |  | 0 |  |  |  |  | 0 |
| 5357 |  |  |  |  | 1 |  |  |  |  | 3 |  |  |  |  | 0 |  |  |  |  | 0 |
| 5359 |  |  |  |  | 0 |  |  |  |  | 0 |  |  |  |  | 0 |  |  |  |  | 0 |
| 5360 |  |  |  |  | 0 |  |  |  |  | 1 |  |  |  |  | 0 |  |  |  |  | 0 |
| 5369 |  |  |  |  | 0 |  |  |  |  | 0 |  |  |  |  | 0 |  |  |  |  | 0 |
| 5370 |  |  |  |  | 0 |  |  |  |  | 0 |  |  |  |  | 0 |  |  |  |  | 0 |
| 5371 |  |  |  |  | 0 |  |  |  |  | 0 |  |  |  |  | 0 |  |  |  |  | 0 |
| 5372 |  |  |  |  | 0 |  |  |  |  | 0 |  |  |  |  | 0 |  |  |  |  | 0 |
| 5373 |  |  |  |  | 0 |  |  |  |  | 0 |  |  |  |  | 0 |  |  |  |  | 0 |
| 5375 |  |  |  |  | 5 |  |  |  |  | 0 |  |  |  |  | 0 |  |  |  |  | 0 |
