# Supplementary material for: Integrated multi-level quality control for proteomic profiling studies using mass spectrometry
Source: BMC Bioinformatics. 2008 Dec 4;9:519. doi: 10.1186/1471-2105-9-519 (PMC2657802; doi:10.1186/1471-2105-9-519)
Supplement: Additional file 5 — Table S3. This file contains a table of summary statistics concerning the mean spectra and CV spectra displayed in Figure 6. [file 1471-2105-9-519-S5.doc]

Table S3. Summary statistics for distribution of mean intensity values and coefficient of variation values. The median is less than the mean in each case indicating a distribution skewed to the left with a longer tail on the right.

|  | **Mean** | **CV (%)** |
| --- | --- | --- |
| Minimum | 0.00 | 9.5 |
| 1st Quartile | 1.83 | 46.0 |
| Median | 5.89 | 118.3 |
| Mean | 12.46 | 162.5 |
| 3rd Quartile | 9.76 | 218.2 |
| Maximum | 293.50 | 612.5 |
